# Accelerated Intermittent Theta Burst Stimulation in Smoking Cessation: Placebo Effects Equal to Active Stimulation When Using Advanced Placebo Coil Technology

**DOI:** 10.3389/fpsyt.2022.892075

**Published:** 2022-05-24

**Authors:** Georgios Mikellides, Panayiota Michael, Lilia Psalta, Artemis Stefani, Teresa Schuhmann, Alexander T. Sack

**Affiliations:** ^1^Department of Cognitive Neuroscience, Faculty of Psychology and Neuroscience, Maastricht University, Maastricht, Netherlands; ^2^Cyprus rTMS Centre, Larnaca, Cyprus; ^3^Department of Psychology, University of Cyprus, Nicosia, Cyprus; ^4^School of Science, University of Central Lancashire, Larnaca, Cyprus; ^5^Department of Developmental Neurosciences, Great Ormond Street Institute of Child Health, University College London, London, United Kingdom; ^6^Department of Psychiatry and Neuropsychology, School for Mental Health and Neuroscience (MHeNs), Brain+Nerve Centre, Maastricht University Medical Centre+ (MUMC+), Maastricht, Netherlands

**Keywords:** smoking cessation, intermittent theta burst stimulation (iTBS), repetitive transcranial magnetic stimulation, provocative smoking cues, placebo effect

## Abstract

Smoking is currently one of the main public health problems. Smoking cessation is known to be difficult for most smokers because of nicotine dependence. Repetitive transcranial magnetic stimulation (rTMS) over the left dorsolateral prefrontal cortex (DLPFC) has been shown to be effective in the reduction of nicotine craving and cigarette consumption. Here, we evaluated the efficacy of accelerated intermittent theta burst stimulation (aiTBS; four sessions per day for 5 consecutive days) over the left DLPFC in smoking cessation, and we investigated whether the exposure to smoking-related cues compared to neutral cues during transcranial magnetic stimulation (TMS) impacts treatment outcome. A double-blind, randomized, controlled study was conducted in which 89 participants (60 males and 29 females; age 45.62 ± 13.42 years) were randomly divided into three groups: the first group received active aiTBS stimulation while watching neutral videos, the second group received active aiTBS stimulation while watching smoking-related videos and the last group received sham stimulation while watching smoking-related videos. Our results suggest that aiTBS is a tolerable treatment. All treatment groups equally reduced cigarette consumption, nicotine dependence, craving and perceived stress. The effect on nicotine dependence, general craving and perceived stress lasted for at least 1 week after the end of treatment. Active aiTBS over the left DLPFC, combined with smoking related cues, is as effective as active aiTBS combined with neutral cues as well as placebo aiTBS in smoking cessation. These findings extend the results of previous studies indicating that TMS therapy is associated with considerably large placebo effects and that these placebo effects may be further increased when using advanced placebo coil technology.

**Clinical Trial Registration:**

www.clinicaltrials.gov, identifier NCT05271175.

## Introduction

Cigarette smoking is one of the foremost causes of preventable disease and premature death (1–5). According to the World Health Organization (WHO), in 2020, 22.3% of the global population used tobacco (6). Nicotine is a highly addictive chemical compound (7) in tobacco and is released directly in the mesolimbic dopamine pathways where reward processing takes place (8). In 2014, 68% of US adult smokers wanted to quit smoking and in 2017, 55.1% of US adult smokers had made an attempt to quit smoking (9–11). However, only a small percentage of adult smokers (7.4%) actually achieved to quit smoking (11). To support smokers in smoking cessation, behavioral, psychological and pharmacological interventions as well as nicotine replacement therapy are some of the most used interventions (12) with medium to low success rates (12, 13). Recently, there has been growing interest in new, alternative, and effective treatments for smoking cessation.

Transcranial magnetic stimulation (TMS) is a non-invasive brain stimulation therapy (14, 15) that delivers magnetic pulses to a brain region, inducing an electric current that can depolarize neurons and induce action potentials (14). Repetitive (r)TMS protocols have been found to have lasting effects on excitability that can either be (generally) inhibitory (1 Hz) or excitatory (10 Hz) in nature by engaging synaptic plasticity mechanisms, such as long-term potentiation (LTP) and long-term depression (LTD) (16). Theta Burst Stimulation (TBS) is a more recent TMS protocol that delivers a comparable number of pulses in a very short time (17, 18). Two different patterns of TBS were developed: intermittent TBS (iTBS) and continuous TBS (cTBS) which generally increases and decreases cortical excitability, respectively (17).

The dorsolateral prefrontal cortex (DLPFC) is a frontal brain region that plays a crucial role in meso-cortico-limbic and serotonergic systems (19) and is involved in executive functions such as inhibitory control, as well as emotion regulation and decision making; processes modified by substance use and dependence (19–21). Mesolimbic dopamine reward circuits and frontoparietal networks are associated with craving and are activated by addictive drugs (22). Exposure to cigarette-related cues has been associated with activation in the DLPFC (23, 24). Smoking related cues provoke activation of these brain circuits of smokers (24, 25). The combination of rTMS with smoking related cues has been found to be more effective compared to the combination of rTMS alone (26).

Several lines of evidence support the efficacy of high frequency (HF)-rTMS over the left DLPFC in the reduction of nicotine craving and cigarette consumption (21, 24, 27) and cue-induced smoking craving (28). A recently published double blind RCT showed that HF-rTMS (20 Hz) over the left DLPFC for 10 daily sessions is effective in reducing cigarette consumption, craving, dependence as well as in improving anxiety and depressing symptoms (29). According to a recent systematic review, multiple target HF-rTMS may be effective in smoking cessation (21). Accelerated TMS (aTMS), is used increasingly in research and clinical practice and has been shown to be as effective as a standard TMS procedure (30–32). Recently, an accelerated, high-dose, iTBS protocol has shown promising results in patients with treatment resistant depression (33).

A growing body of research highlights the importance of determining the efficacy of TMS in neuropsychiatric disorders using randomized controlled trials (RCT) with placebo-controlled groups. Placebo effects in TMS are a very common phenomenon (34–37) and can have a big influence on the results of a study (38). Several studies indicated that the placebo effect may be a component of the therapeutic response to rTMS in neuropsychiatric disorders like major depressive disorder, and stroke rehabilitation (35, 37).

Considering current knowledge of the efficacy of iTBS in substance use disorders, we investigated in a double-blind randomized control trial efficacy of four iTBS sessions per day during five consecutive days over the left DLPFC in smoking cessation, using the Cool-B65 Active/Placebo (A/P) coil, an advanced coil that is designed to support true “double blinded” clinical trials. Moreover, we wanted to investigate whether the exposure to smoking-related cues during the rTMS treatment, compared to neutral cues impacts cigarette craving. We hypothesized that 20 sessions of accelerated theta burst simulation over the left DLPFC while exposed to smoking-related cues, would reduce cigarette consumption and cigarette cravings, accompanied by reduced stress and motivation to quit smoking to a greater extent than active stimulation combined with neutral cues and sham stimulation with smoking-cues.

## Materials and Methods

### Participants

One hundred fifty-nine cigarettes smokers, who wanted to quit smoking, were recruited *via* internet advertisements and printed flyers in the period of April 2019 to December 2020 in Cyprus. Potential participants were screened in a short telephone interview where a total of 104 participants were eligible to participate. Inclusion criteria were the following: (a) aged 18–70, (b) native or fluent Greek speaker. Exclusion criteria were the following: (a) mental objects or implants in the brain, skull or near head (e.g., pacemakers, metal plates), (b) past or current of diagnosis of neurological or psychiatric disorder, (c) use of psychiatric medication, (d) past or current drug or alcohol abuse, other than nicotine, (e) use of IQOS (“I Quit Original Smoking”) or electronic cigarettes (e-cigarettes). A total of 89 participants were included in the final analysis (60 males and 29 females; age 45.62 ± 13.42 years), excluding dropouts (*n* = 15). The minimum number of participants required was determined by an a priori power analysis where at least a sample size of 100 participants was suggested. [^*^Measures that suggested this sample size were determined by the mixed model, a small to medium effect size (0.4), at an alpha level of probability of 0.05]. The experiment was carried out in the Cyprus rTMS Center in Larnaca, Cyprus. This study was approved by the Cyprus National Bioethics Committee and written informed consent was obtained from all participants (EEBK/EΠ/2019/08).

### Experimental Design

A multi-arm parallel group, double-blind, randomized, controlled study was conducted in which participants were randomly divided into three groups: the first group received active iTBS stimulation while watching neutral videos (TMS&N group), the second group received active iTBS stimulation while watching smoking-related videos (TMS&S group) and the last group received sham stimulation while watching smoking-related videos (Sham group). The Latin square design was used for the randomization. Both participants and the investigator who applied the rTMS and administered the self-reported measurements to the participants were blinded to the treatment condition. A second investigator was not blinded to the procedures to be able to set-up the appropriate stimuli. Four iTBS sessions (active or sham) were administrated every day, with 30 min break between them over a 5-day period. Both active iTBS stimulation and sham stimulation were applied over the left DLPFC.

### RTMS Procedure

Stimulation was performed using a MagPro X100 (MagVenture, Farum, Denmark) and a figure-of-eight coil (Coil Cool-B65 A/P) for both active and sham stimulation. The Cool-B65 Active/Placebo (A/P) coil is designed to support true “double blinded” clinical trials as it can produce active and placebo stimulation by flipping the coil and can mimic a tapping sensation during placebo condition (39) (see The MagVenture Cool-B65 Active/Placebo (A/P) Coil in Supplementary Material 1 for additional information).

Before the first session, the resting Motor Threshold (rMT) was determined by placing the coil over the left primary motor cortex (40) (see Resting Motor Threshold (rMT) in Supplementary Material 2 for additional information). Stimulation was performed at 100% of rMT. Two experimenters were in the treatment room with the participant. The TMS operator (blinded experimenter) avoided watching the video while it was playing to remain blinded to the procedure and was only looking into the patients' direction. The videos were played by the second researcher.

In both active and sham conditions, an accelerated iTBS (aiTBS) treatment (four sessions with 30 min break between them) was administered daily for a 5-day period over the left DLPFC. Beam_F3 Locator software was used to locate the left DLPFC (41) (see Beam_F3 Locator Software in Supplementary Material 3 for additional information). The stimulation coil was placed at a 45° angle of the midline. iTBS was administrated at 5 Hz and each session included 20 trains with 8 s inter train interval (10 pulses per train at 50 Hz). A total number of 600 pulses was given per session.

### Data Collection and Measurements

Demographic information as well as smoking-habits profile information were collected (Table 1). Participants were asked to report the number of cigarettes usually smoked during a day as well as the type of cigarettes, years of smoking and whether they ever quit smoking and if yes, how many times, to record smoking habits (Table 1).

**Table 1:** Demographic and smoking-related characteristics of (*N* = 89) participants.

**Characteristics**	**TMS&N group**	**TMS&S group**	**Sham group**	***p*-Values**
	***n* = 29**	***n* = 30**	***n* = 30**	
**Demographic**				
Age (year)	46.52 ±13.05	42.93 ± 14.42	47.43 ± 12.72	0.395^a^
Gender (M/F)	22/7	20/10	18/12	0.427^b^
Education (year)	14.07 ± 3.95	14.43 ± 30.77	13.60 ± 3.27	0.681^a^
Occupation^*^				0.167^b^
Private employee	13 (14.61%)	19 (21.35%)	22 (24.72%)	
Public employee	7 (7.87%)	4 (4.49%)	1 (1.12%)	
Self-employed/Freelancer	5 (5.62%)	1 (1.12%)	4 (4.49%)	
Unemployed	2 (2.25%)	1 (1.12%)	0 (0%)	
Retired	2 (2.25%)	4 (4.49%)	3 (3.37%)	
Student	0 (0%)	1 (1.12%)	0 (0%)	
**Smoking-related**				
Cigarettes per day	27.55 ± 15.37	26.83 ± 12.86	30.00 ± 13.38	0.654^a^
Types of cigarettes^*^				0.184 ^b^
Normal	16 (17.98%)	25 (28.09%)	24 (26.97%)	
Hand-rolled	10 (11.24%)	5 (5.62%)	5 (5.62%)	
Cigarillos	1 (1.12%)	0 (0%)	0 (0%)	
Mixed	2 (2.25%)	0 (0%)	1 (1.12%)	
Years of smoking	23.18 ± 9.82	23.13 ± 13.58	28.73 ± 12.21	0.125^a^
If ever quitted^*^				0.899^b^
No	9 (10.11%)	10 (11.24%)	11 (12.36%)	
Yes	20 (22.5%)	20 (22.5%)	19 (21.3%)	
How many times quitted	0.90 ± 0.77	1.00 ± 1.11	1.20 ± 1.56	0.614^a^

*Data are means ± standard deviation*.

* *n (%)*.

a *One-way ANOVA*.

b *Pearson chi-square test*.

*TMS, transcranial magnetic stimulation*.

### Smoking-Related and Neutral Video Cues

During the rTMS treatment, participants were instructed to pay attention to videos that were presented on a monitor (Height: 20 cm; Width: 35 cm) placed opposite the treatment chair. Two different forms of videos were used (smoking related videos e.g., a person smoking cigarette in a restaurant and neutral videos e.g., a man cleaning his shoes) in order to elicit craving at the time of stimulation. Each video was presented for approximately 3 min during the stimulation.

### Primary Measures

Cigarette consumption: (a) Self-reported nicotine consumption: Participants had to daily record the number of cigarettes smoked from the completion of the four sessions until their next treatment visit. Participants were asked not to smoke during the breaks of the four daily rTMS sessions; (b) Carbon monoxide (CO)- evaluated nicotine consumption: CO levels were measured using the piCO Smokerlyzer breath carbon monoxide meter device.

Nicotine dependence: Fagerström test for Nicotine Dependence (FTND) (42) is a short, self-report measure that assesses nicotine dependence. It contains six questions, and the total score is calculated as a sum of these six questions. The total scores of the questionnaire vary from 0 to 10, with lower scores indicating lower dependence on nicotine. This scale has been used previously in Cypriot samples and has been translated into Greek, showing good internal consistency (43, 44).

Craving: (a) Momentary Craving: The Visual Analog Scale (VAS) is a psychometric measurement instrument that measures symptom severity on a continuous scale (45). We used the VAS to assess smoking craving by asking participants to respond to the question “How much do you want to smoke right now?”, on a scale from 0 “no craving” to 100 “most craving ever experienced”; (b) General Craving: Tobacco Craving Questionnaire–Short Form (TCQ-SF) (46) is a self-report measure that assesses tobacco craving in four dimensions: emotionality, craving in anticipation of relief from withdrawal or negative mood; expectancy, craving in anticipation of positive outcomes from smoking; compulsivity, craving in anticipation of an inability to control tobacco use; and purposefulness, craving coupled with intention and planning to smoke. Each factor scale contains three items. TCQ-SF items were rated on a Likert scale of 1 (strongly disagree) to 7 (strongly agree). Total scores vary from 12 to 84, by summing the 12 items and the scores for each factor scale vary from 3 to 21 by summing the three items in each factor scale. A high score indicates high tobacco craving. We translated the TCQ-SF into Greek using the forward and backward-translation procedure (Cronbach's α = 0.90, see Cronbach's alpha in Supplementary Material 4 for additional information).

### Secondary Measures

Perceived Stress: Perceived Stress Scale-4 (PSS-4) (47) is a self-report measure that is used to assess psychological stress. The original PSS comprises 14 items (PSS-14) with two (negative and positive) subscales. We here used the shorter version with four items (PSS-4) that were rated on a Likert scale, ranging from 0 to 4, with those on the positive subscale scored in reverse and the total score was calculated as a sum of these items. The scores vary from 0 to 16, with a higher score indicating higher perceived stress.

Motivation to quit smoking: Participants were asked to estimate how motivated they were to quit smoking from 0 to 100%.

Adverse events: Participants were asked to daily report the adverse events they may have had experienced.

(For the time points of each measurement, see Table 2).

**Table 2:** Overview of data collection time points.

**Measurements**	**Time points**
**Primary measures**	
Self-reported cigarette consumption	i Baseline
	ii AfterDay1
	iii AfterDay2
	iv AfterDay3
	v AfterDay4
Carbon monoxide (CO)- evaluated nicotine consumption	Prior to each rTMS session
Fagerström test for nicotine dependence (FTND)	i Baseline
	ii End of the treatment
	iii 1 week follow up
The Visual Analog Scale (VAS)	Prior to and post each rTMS session
Tobacco Craving	i Baseline
Questionnaire–Short Form	ii End of treatment
(TCQ-SF)	iii1 week follow up
**Secondary measures**	
Perceived Stress Scale-4 (PSS-4)	i Baseline
	ii End of the treatment
	iii 1 week follow up
Motivation to quit smoking	i Baseline
	ii End of the treatment
	iii 1 week follow up
Adverse events	After each treatment day

### Data Analysis

SPSS software version 27.0 was used for the statistical analysis of the data (IBM corporation, Endicott, New York). We calculated the mean score of the 8 VAS scores and 4 CO scores of each day. A one-way ANOVA and Pearson chi-square test were used to test for differences in baseline demographic and smoking-related variables and rMT scores between the three groups. Mixed factorial ANOVAs were conducted to investigate the effect of both the within factor (Time) and the between factor (Group: TMS-N group, TMS-S group, Sham group). The dependent variables used for each model were: cigarette consumption, nicotine dependence, craving and perceived stress. Greenhouse–Geisser and Huynd-Feldt degree of freedom corrections were applied to correct for the non-sphericity the data. *Post hoc* comparisons using paired-samples *t*-test were used to evaluate the significance of mean change in cigarette consumption, nicotine dependence, craving and perceived stress at different timepoints. Non-parametric tests were used as the variable *Motivation to quit smoking* was not normally distributed at all time-point assessments. Non-parametric Wilcoxon signed-rank tests were conducted to evaluate the significance of mean change in *Motivation to quit smoking* scores at different time points for each Group separately and non-parametric Kruskal–Wallis *H* tests were conducted to compare the mean scores of motivation to quit of the three Groups at different timepoints. Pearson chi-square test was used to test for differences in adverse events between the active TMS and sham TMS. Finally, a Pearson correlation analysis was applied to correlate a subjective measure (self-reported) with an objective measure (CO) of nicotine consumption. A significance level was set at α = 0.05 for all analyses.

## Results

### Baseline Characteristics

**Figure 1:**
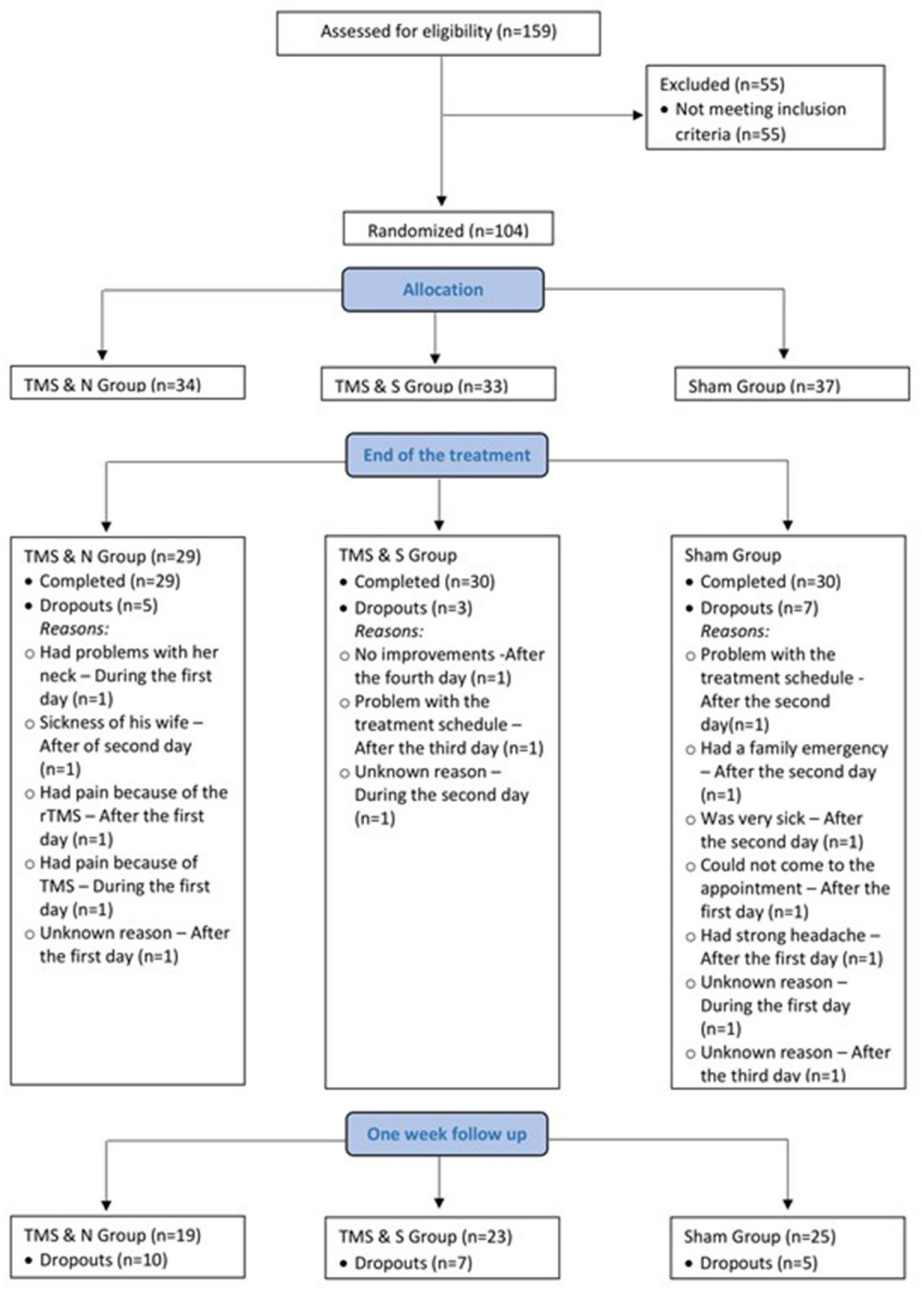
Flow chart of the selection procedure.

Eight-nine participants completed the entire treatment program (60 males and 29 females; age 45.62 ± 13.42 years; see Enrollment in Supplementary Material for enrollment information and Figure 1 for study recruitment flow diagram). Participant demographics and smoking-related variables are listed in Table 1. Analysis showed that the three groups did not differ significantly in demographic or smoking-related characteristics (all *p* > 0.05).

### Primary Outcomes

#### Self-Reported Nicotine Consumption

**Figure 2:**
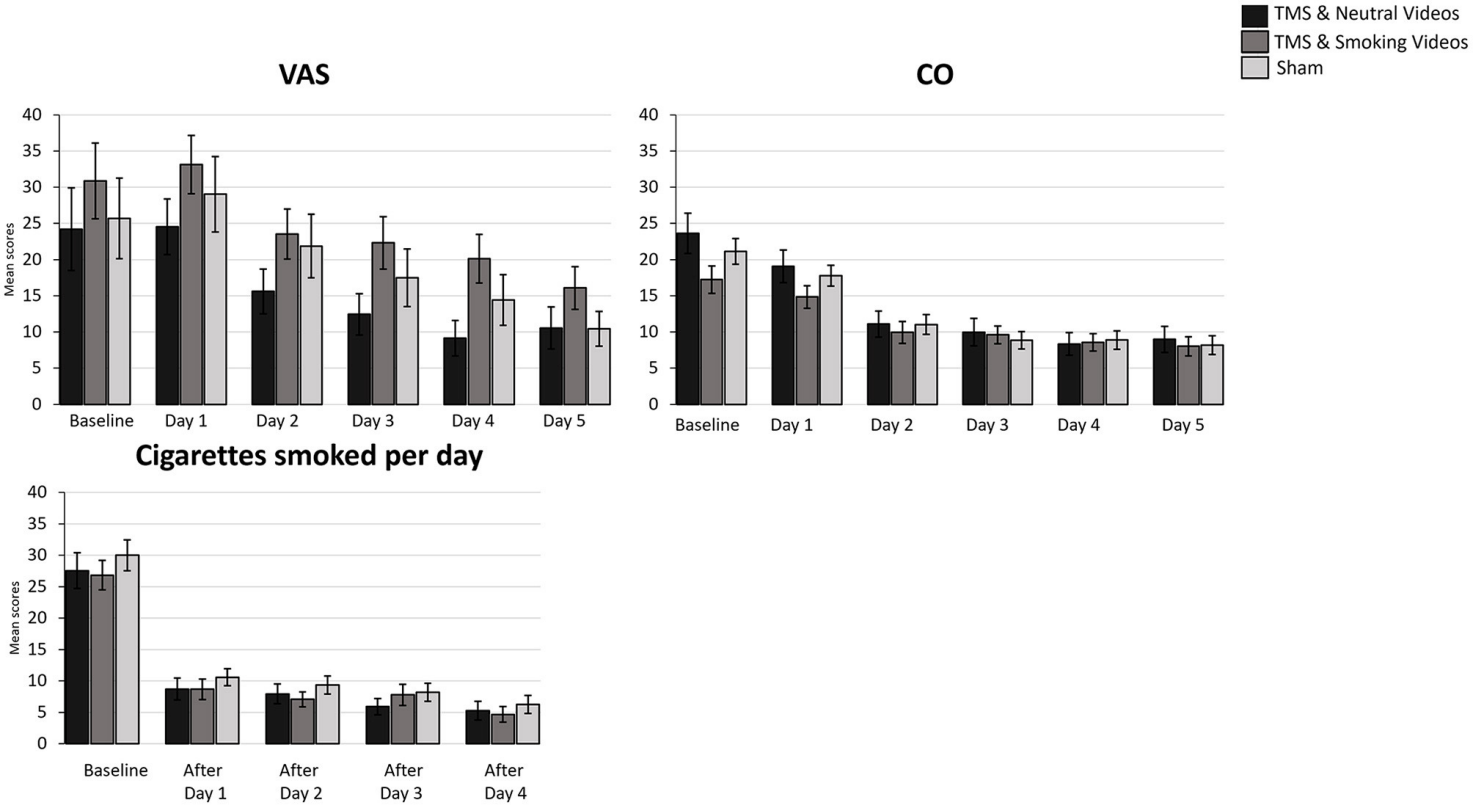
Bar graphs showing difference in mean scores of VAS, CO, Cigarettes smoked per day over time. Data are presented as mean ± SEM.

A 5 (Time: Baseline, AfterDay1, AfterDay2, After Day3, AfterDay4) × 3 (Group: TMS&N group, TMS&S group, Sham group) mixed factorial ANOVA was conducted for the analysis of the number of cigarettes smoked per day. Mauchly's test indicated that the assumption of sphericity had been violated, χ^2^(9) = 167.688, *p* = 0.00, therefore degrees of freedom were corrected using Greenhouse-Geisser of sphericity (ε = 0.470). There was a statistically significant main effect of Time, *F*(1.879, 142.840) = 166.548, *p* < 0.0001, η*p*^2^ = *0.687*, suggesting a significant decrease in the number of cigarettes smoked per day over time. However, there was no significant effect of Type of Group, *F*(2, 76) = 0.363, *p* = 0.697, η*p*^2^ = *0.009* (Figure 2, see Supplementary Table 1 for means and standard deviations). The interaction effect between Time and Group was not statistically significant, *F*(3.759, 142.840) = 0.414, *p* = 0.787, η*p*^2^ = *0.011*. *Post hoc* comparisons using paired-samples *t*-test were used to evaluate the significance of mean change in the number of cigarettes smoked per day at different time points (Table 3). Results indicate that mean scores were statistically significantly lower over time in all the comparisons, except of the pair AfterDay1 vs. AfterDay2, where no statistically significantly changes were found.

**Table 3:** Results of paired sample *t*-test for the number of cigarettes smoked per day.

	**Mean change**	**SD**	***t-*Value**	***p*-Value**
Pair 1: Baseline vs. AfterDay1	−19.13	11.89	14.731	**<0.0001**
Pair 2: Baseline vs. AfterDay2	−20.48	11.73	16.188	**<0.0001**
Pair 3: Baseline vs. AfterDay3	−21.20	12.83	14.962	**<0.0001**
Pair 4: Baseline AfterDay4	−22.93	12.89	16.208	**<0.0001**
Pair 5: AfterDay1 vs. AfterDay2	−1.14	5.35	1.940	0.056
Pair 6: AfterDay1 vs. 1 AfterDay3	−2.13	7.44	2.597	0.011
Pair 7: AfterDay1 vs. AfterDay4	−3.82	6.85	5.051	**<0.0001**
Pair 8: AfterDay2 vs. AfterDay3	−1.09	4.90	2.006	0.048
Pair 9: AfterDay2 vs. AfterDay4	−2.84	5.09	5.050	**<0.0001**
Pair 10: AfterDay3 vs. AfterDay4	−1.74	4.64	3.363	**0.001**

*Paired sample t-test; p < 0.05. Significant after Bonferroni correction in bold*.

#### CO-evaluated Nicotine Consumption

A 6 (Time: Baseline, Day1, Day2, Day3, Day4, Day5) × 3 (Group: TMS&N group, TMS&S group, Sham group) mixed factorial ANOVA was conducted for the analysis of CO scores. Mauchly's test indicated that the assumption of sphericity had been violated, χ^2^(14) = 340.631, *p* = 0.00, therefore degrees of freedom were corrected using Greenhouse-Geisser of sphericity (ε = 0.368). The interaction effect between Time and Group was not statistically significant, *F*(3.678, 154.484) = 1.964, *p* = 0.109, η*p*^2^ = *0.045*. There was a statistically significant main effect of Time, *F*(1.839, 154.484) = 82.421, *p* < 0.0001, η*p*^2^ = *0.495*, suggesting a significant decrease in CO scores over time. However, there was no significant effect of Group, *F*(2, 84) = 0.589, *p* = 0.557, η*p*^2^ = *0.014* (Figure 2, see Supplementary Table 1 for means and standard deviations).

#### Nicotine Dependence

**Figure 3:**
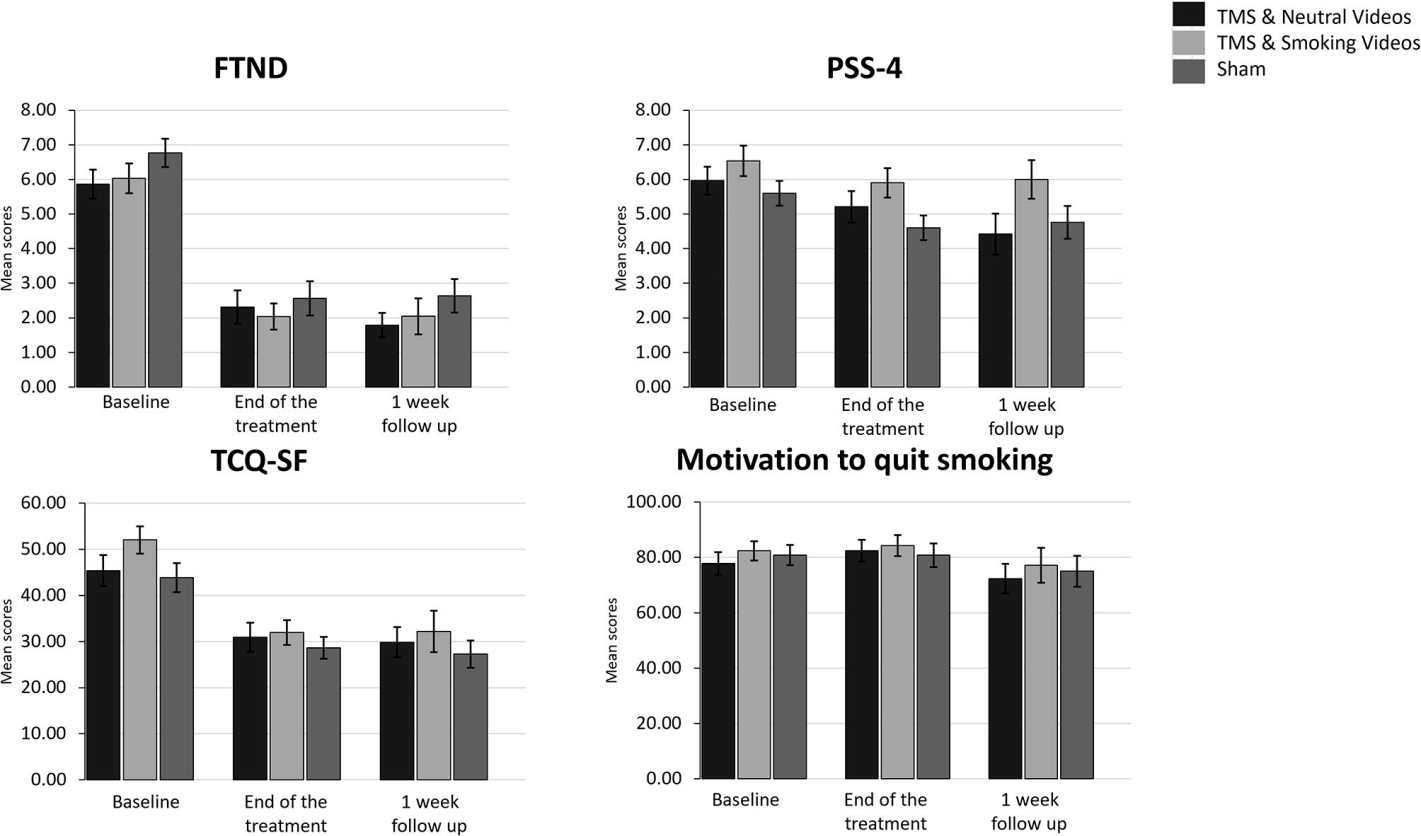
Bar graphs showing difference in mean scores of FTND, PSS-4, TCQ-SF and Motivation to quit smoking over time. Data are presented as mean ± SEM.

A 3 (Time: Baseline, End of treatment, 1 week follow up) × 3 (Group: TMS&N group, TMS&S group, Sham group) mixed factorial ANOVA was conducted as measured by the FTND. Mauchly's test indicated that the assumption of sphericity had been violated, χ^2^(2) =11.064, *p* = 0.004, therefore degrees of freedom were corrected using Huynh-Feldt of sphericity (ε = 0.911). The interaction effect between Time and Group was not statistically significant, *F*(3.642, 116.549) = 0.095, *p* = 0.978, η*p*^2^ = *0.003*. There was a statistically significant main effect of Time, *F*(1.821, 116.549) = 119.672, *p* < 0.0001, η*p*^2^ = *0.652*, suggesting a significant decrease in nicotine dependence over time. However, there was no significant effect of Group, *F*(2, 64) = 1.784, *p* = 0.176, η*p*^2^ = *0.053* (Figure 3, see Supplementary Table 1 for means and standard deviations). *Post-hoc* paired sample *t*-tests were used to evaluate the significance of mean change in FTND scores at different time points (Table 4). Results indicate that mean scores were statistically significantly lower at the End of treatment and at 1 month follow up compared to the baseline, however, no statistically significantly changes were found between the scores at the End of treatment compared to the scores at 1 week follow up.

**Table 4:** Results of paired sample *t*-test for the three self-reported measures.

	**Mean change**	**SD**	***t-*Value**	***p*-Value**
**FTND**				
Pair 1: Baseline vs. End of treatment	−3.92	2.570	14.379	**<0.0001**
Pair 2: Baseline vs. 1 week follow up	−3.82	2.57	12.170	**<0.0001**
Pair 3: End of treatment vs. 1 week follow up	0.12	1.79	–**0.5**44	0.588
**TCQ-SF**				
Pair 1: Baseline vs. End of treatment	−16.59	19.60	7.988	**<0.0001**
Pair 2: Baseline vs. 1 week follow up	−15.13	20.56	6.026	**0.010**
Pair 3: End of treatment vs. 1 week follow up	1.72	14.09	–**0.9**97	0.323
**PSS-4**				
Pair 1: Baseline vs. End of treatment	–**0.7**9	1.95	3.861	**<0.0001**
Pair 2: Baseline vs. 1 week follow up	−1.07	2.47	3.561	**0.001**
Pair 3: End of treatment vs. 1 week follow up	–**0.1**8	2.24	0.654	0.516

*Paired sample t-test; p < 0.05. Significant after Bonferroni correction in bold*.

#### Momentary Craving

A 6 (Time: Baseline, Day 1, Day 2, Day3, Day 4, Day 5) × 3 (Group: TMS&N group, TMS&S group, Sham group) mixed factorial ANOVA was conducted for the analysis of VAS scores. Mauchly's test indicated that the assumption of sphericity had been violated, χ^2^(14) = 160.748, *p* = 0.00, therefore degrees of freedom were corrected using Greenhouse-Geisser of sphericity (ε = 0.539). The interaction effect between Time and Group was not statistically significant, *F*(5.389, 231.740) = 0.400, *p* = 0.861, η*p*^2^ = *0.009*. There was a statistically significant main effect of Time, *F*(2.695, 231.740) = 25.667, *p* < 0.0001, η*p*^2^ = *0.230*, suggesting a significant decrease in VAS scores over time. However, there was no significant effect of Group, *F*(2, 86) = 1.511, *p* = 0.226, η*p*^2^ = *0.034* (Figure 2, see Supplementary Table 1 for means and standard deviations).

#### General Craving

A 3 (Time: Baseline, End of treatment, 1 week follow up) × 3 (Group: TMS&N group, TMS&S group, Sham group) mixed factorial ANOVA was conducted as measured by the TCQ-SF. Mauchly's test indicated that the assumption of sphericity had been violated in both situations, χ^2^(2) = 11.572, *p* = 0.003, therefore degrees of freedom were corrected using Huynh-Feldt of sphericity (ε = 0.905). The interaction effect between Time and Group was not statistically significant, *F*(3.620, 115.845) = 1.320, *p* = 0.269, η*p*^2^ = *0.040*. There was a statistically significant main effect of Time, *F*(1.810, 115.845) = 32.881, *p* < 0.0001, η*p*^2^ = *0.339*, suggesting a difference in tobacco craving over time. However, there was no significant effect of Group, *F*(2, 64) = 2.289, *p* = 0.110, η*p*^2^ = *0.067* (Figure 3, see Supplementary Table 1 for means and standard deviations). *Post-hoc* paired sample *t*-tests were used to evaluate the significance of mean change in TCQ-SF scores at different time points (Table 4). Results indicate that mean scores were statistically significantly lower at the End of treatment and at 1 month follow up compared to the baseline, however, no statistically significantly changes were found between the scores at the End of treatment compared to the scores at 1 week follow up.

### Secondary Outcomes

#### Perceived Stress

A 3 (Time: Baseline, End of treatment, 1 week follow up) × 3 (Group: TMS&N group, TMS&S group, Sham group) mixed factorial ANOVA was conducted as measured by PSS-4. The interaction effect between Time and Group was not statistically significant, *F*(4, 128) = 1.132, *p* = 0.344, η*p*^2^ = *0.034*. There was a statistically significant main effect of Time, *F*(2, 128) = 9.398, *p* < 0.0001, η*p*^2^ = *0.128*, suggesting a significant decrease in perceived stress over time. However, there was no significant effect of Group, *F*(2, 64) = 1.415, *p* = 0.250, η*p*^2^ = *0.042* (Figure 3, see Supplementary Table 1 for means and standard deviations). *Post-hoc* paired sample *t*-tests were used to evaluate the significance of mean change in PSS-4 scores at different time points (Table 4). Results indicate that mean scores were statistically significantly lower at the End of treatment and at 1 month follow up compared to the baseline, however, no statistically significantly changes were found between the scores at the End of treatment compared to the scores at 1 week follow up.

#### Motivation to Quit Smoking

Wilcoxon signed-rank tests yielded no statistically significantly changes, expect of the pair End of treatment vs. 1 week follow up of the TMS& N Group (*Z* = −2.392, *p* = 0.017) where scores at 1 week follow up (Mean = 72.37, SD = 23.41) were statistically significantly lower compared to the scores at the End of treatment (Mean = 82.41, SD = 20.59). Also, Kruskal–Wallis *H* tests showed that there were no statistically significant differences in Motivation scores between the different Groups in the baseline, χ^2^(2) = 0.646, *p* = 0.724, at the End of treatment, χ^2^(2) = 0.202, *p* = 0.904 and at the 1 week follow up, χ^2^(2) = 0.810, *p* = 0.667 (Figure 3, see Supplementary Table 1 for means and standard deviations).

#### Adverse Events

Eleven participants (37.93%) of the TMS-N Group, five participants (16.67%) of the TMS&S group and seven participants (23.33%) of the Sham group reported mild adverse events. There were no statistically significant differences between Active and Sham TMS in terms of adverse events as determined by Pearson chi-square test (*p* = 0.574). The most frequent adverse events were mild headache and sleepiness (Table 5). No severe adverse events such as seizure or mania have been reported in the study.

**Table 5:** Adverse events of (*N* = 23) participants, *n* (%).

**Adverse events**	**Active TMS**	**Sham TMS**	**Total**
Mild headache	6 (26.1%)	1 (4.3%)	7 (30.4%)
Sleepiness	3 (13%)	2 (8.7%)	5 (21.7%)
Insomnia	1 (4.3%)	1 (4.3%)	2 (8.7%)
Tension	1 (4.3%)	1 (4.3%)	2 (8.7%)
Nausea	0 (0%)	1 (4.3%)	1 (4.3%)
Numbness on stimulation site	1 (4.3%)	0 (0%)	1 (4.3%)
Lightheadedness	1 (4.3%)	0 (0%)	1 (4.3%)
Coughiness	1 (4.3%)	0 (0%)	1 (4.3%)
Numbness on stimulation site & Forgetfulness	0 (0%)	1 (4.3%)	1 (4.3%)
Numbness on stimulation site & Sleepiness	1 (4.3%)	0 (0%)	1 (4.3%)
Mild headache & Sleepiness	1 (4.3%)	0 (0%)	1 (4.3%)
**Total adverse events**	**16 (69.6%)**	**7 (30.4%)**	**23 (100%)**

*TMS, transcranial magnetic stimulation*.

#### Correlations Between Self-Reported and CO-measured Nicotine Consumption

A Pearson correlation analysis was applied to correlate self-reported and CO-measured nicotine consumption. Results showed a significant positive correlation between the two variables in all timepoints (see Supplementary Table 2).

## Discussion

The current study investigated the efficacy of a rapid accelerated iTBS therapy (four sessions per day for five consecutive days) combined with smoking related cues in smoking cessation. We hypothesized that an active TMS group that is exposed to smoking related videos during stimulation (TMS&S group) shows more improvement with regard to reducing their cigarette consumption and smoking craving compared to the group that receives sham stimulation while watching smoking-related videos (sham group), and to the group receiving active TMS while watching neutral videos (TMS&N group).

In contrast to these expectations, we however found that all conditions, including sham stimulation, were equally effective in reducing cigarette consumption, CO levels, smoking craving and nicotine dependence. Contrary to our expectations and to what is reported in the literature, active TMS combined with smoking related cues was not more effective than active TMS combined with neutral cues, not sham stimulation.

Most interestingly was the fact that our TMS intervention was highly effective in facilitating smoking cessation. Our participants in the active TMS conditions showed 80.7 and 82.59% decrease in cigarette consumption in TMS &N Group and TMS&S group respectively, and 56.38 and 47.59% reduction in nicotine craving in TMS &N Group and TMS&S group respectively. The number of cigarettes smoked per day was statistically significantly lower over time, from the baseline to the End of treatment of the fifth day. These results are consistent with previous TMS trials, which show that rTMS can significantly reduce cigarette consumption and nicotine craving (21, 24, 26). Surprisingly, our advanced placebo coil technology condition specifically designed to support true “double blinded” clinical trials showed to be equally effective in treating smoking cessation. Our participants in Sham group showed 79.1% decrease in cigarette consumption and 59.34% reduction in nicotine craving. A similar reduction in cigarette consumption was found in a recent RCT, where the reduction in the active group was 76.19% (27), although, contrary to our findings, a much smaller reduction in cigarette consumption was found in the sham group (35.29%). Similarly, participants in all conditions showed huge reductions in CO scores (TMS&N group: 62.01%, TMS&S group: 53.42%, Sham group: 61.29%).

We were thus able to show, that, especially when using such an advanced double blind placebo stimulation technology, the placebo effect of TMS in clinical context can be considerably large and even equal to the effect achieved with active TMS stimulation. Placebo effects in TMS are known to be playing a certain role on the clinical results obtained with TMS and have been documented before (35–38). There are several factors that contribute to the enhancement of placebo effect in rTMS studies (38, 48). A systematic review and meta-analysis by Razza et al. (37) evaluated the efficacy of rTMS for depression using data from a sham group of 61 RCTs, concluding that placebo effect sizes in depression trials are rather large (g = 0.8). Previous studies also demonstrated that placebo effects may be a component of the therapeutic response to rTMS (35, 37). The placebo effect was also shown to be larger in more intense TMS protocols [HF rTMS (48)] and especially accelerated protocols (49).

We therefore support that several specific factors not directly associated with rTMS treatment have contributed to the enhanced placebo effect found in the present study. First, our participants were highly motivated to quit smoking. Our data clearly indicate that already at day 1 and 2 during the treatment cycle, a strong effect of both, active and placebo TMS, was revealed. The timeline of these effects indicate that this is likely driven more by the motivation and expectation of our participants rather than by actually induced neuroplastic changes. Second, we used an intensive and state-of-the art TMS design, applying accelerated TMS with multiple sessions per day using theta burst stimulation sequences. It has been shown before that placebo effects scale with the intensity and complexity of the used TMS technology (48, 49). Finally, we used an advanced placebo coil technology capable of creating a true double blind clinical trial and an undistinguishable experience for each participant whether or not to be in a placebo or active stimulation condition. Unlike previous TMS studies, we did not use a simple coil tilting procedure (50), or a standard sham coil (51) to achieve our placebo condition. Instead, we used a novel and advanced placebo coil technology capable of mimicking not only the visual and auditory experience of active TMS, but also the somatosensory skin sensation using a low intensity current stimulator built into the A/P coils and a pair of surface electrodes placed just below the hairline on the scalp of each participant. These factors likely contributed to the fact that we do find our accelerated TMS intervention to be highly effective in reducing cigarette consumption and smoking craving, but not significantly more effective than placebo. The actual effect of our active rTMS had to show statistically to be on top of the highly effective placebo condition, which turned out to be not the case in our trial due to the factors mentioned above.

Additionally, our results demonstrated a statistically significant difference in perceived stress over time. However, due to the absence of a significant effects of the Group and the interaction effect between Time and Group, these results are inconclusive regarding the efficacy of active TMS in reducing perceived stress. Nevertheless, previous findings have shown that left DLPFC is a principal target of noninvasive brain stimulation techniques in regulating stress-related cognitive processes (52). It was reported in the literature that perceived stress may be a barrier to smoking cessation (53), and thus further investigation on the association of perceived stress and smoking cessation during rTMS treatment is required.

The follow up assessment proved that these positive effect in nicotine dependence and perceived stress, as measured by FTND, TCQ-SF and PSS-4, lasts at least 1 week after the End of treatment. The findings of this study have to be seen in light of some limitations. Firstly, we did not measure self-reported cigarette consumption after the fifth day of treatment and during the 1-week follow up. Another potential limitation is the absence of a fourth group receiving sham stimulation while watching neutral videos. Finally, we did not use any formal assessment of blinding efficacy.

Although future RCTs are necessary to validate these conclusions, the present study highlights the importance of placebo effects and the role of specific placebo coil technologies in evaluating the efficacy of TMS in any psychiatric and psychological contexts. This could be used to further improve the administration of TMS based interventions, both for designing better placebo conditions in clinical trials, as well as for utilizing TMS placebo for enhancing coping and other psychological strategies of patients during rTMS treatment (48).

## Conclusion

Our findings show that active aiTBS combined with smoking related cues, is as effective as active aiTBS combined with neutral cues as well as placebo aiTBS in smoking cessation. These findings extend the results of previous studies indicating that rTMS therapy is associated with considerably large placebo effects and that these placebo effects may be further increased when using advanced placebo coil technology. These beneficial effects in reducing cigarette consumption and craving for smoking in this and previous studies are likely a combination between the active rTMS effect and the placebo TMS effect. Future RCTs using advanced placebo coil technology are needed to confirm these results. Finally, future studies should emphasize on how to minimize placebo effect on TMS treatment.

## Data Availability Statement

The original contributions presented in the study are included in the article/Supplementary Material, further inquiries can be directed to the corresponding author.

## Ethics Statement

The studies involving human participants were reviewed and approved by Cyprus National Bioethics Committee (EEBK/EΠ/2019/08). The patients/participants provided their written informed consent to participate in this study.

## Author Contributions

GM: conceptualization, methodology, formal analysis, data curation, writing—original draft, and project administration. PM: formal analysis, data curation, and writing—original draft. LP: formal analysis, data curation, and writing—review and editing. AS: conceptualization, methodology, participant recruitment, data collection, formal analysis, data curation, and writing—review and editing. TS: supervision and writing—review and editing. ATS: conceptualization, supervision, and writing—review and editing, and project administration. All authors agree with the contents of the manuscript and were fully involved in the study and preparation of the manuscript and have read the final version of the manuscript and have approved the submission.

## Conflict of Interest

The authors declare that the research was conducted in the absence of any commercial or financial relationships that could be construed as a potential conflict of interest.

## Publisher's Note

All claims expressed in this article are solely those of the authors and do not necessarily represent those of their affiliated organizations, or those of the publisher, the editors and the reviewers. Any product that may be evaluated in this article, or claim that may be made by its manufacturer, is not guaranteed or endorsed by the publisher.
