# Supplementary material for: Accelerated Intermittent Theta Burst Stimulation in Smoking Cessation: Placebo Effects Equal to Active Stimulation When Using Advanced Placebo Coil Technology
Source: Front Psychiatry. 2022 May 24;13:892075. doi: 10.3389/fpsyt.2022.892075 (PMC9170940; doi:10.3389/fpsyt.2022.892075)
Supplement: Supplementary file 1 [file Data_Sheet_1.docx]

Supplementary Material

# The MagVenture Cool-B65 Active/Placebo (A/P) coil

The Cool-B65 Active/Placebo (A/P) coil is designed to support true “double blinded” clinical trials as it can produce active and placebo stimulation by flipping the coil and can mimic a tapping sensation during placebo condition (1). Using a randomization code, the TMS operator obtains information from the MagPro, which shows either “Flip Coil” or “Coil Ready”. Thus, the TMS operator is blinded to the treatment condition.

During the placebo condition, the coil produces the same sound and only a very small amount of current is induced in tissue, and thus no skin-sensation is present. To do so, a low intensity current stimulator is built into the A/P coils and a pair of surface electrodes are placed just below the hairline on the scalp of every participant. Using the surface electrodes, the participant has a similar skin-sensation as in the real active condition.

# Resting Motor Threshold (rMT)

The rMT is the amount of machine output (intensity) required to elicit a motor-evoked potential (MEP) in 50% of all attempts (2). There were no statistically significant differences between the three groups in terms of rMT as determined by a one-way ANOVA, F(2,86)=1.060, p=.351 (TMS-N group: 44.73 ± 55.41; TMS-S group: 44.84 ± 57.62, sham group: 42.69 ± 55.25).

# Beam_F3 Locator Software

Beam_F3 Locator software was used to locate the left DLPFC. This locator software is an efficient and accurate method to mark the F3 position according to the 10-20 EEG system (3). Three main measurements are required for the determination of the F3 position: nasion to inion, tragus to tragus and head circumference.

# Cronbach's alpha

Cronbach's alpha measure was used to assess the internal consistency ("reliability") of the three self-reported questionnaires (FTND, TCQ-SF, PSS-4). Data analysis showed that our version of TCQ-SF had excellent internal consistency (Cronbach’s α=.90). Also, results showed that FTND and PSS-4 had internal consistency of Cronbach’s α=.65 and α=.62, respectively.

# Enrollment

A total of 159 cigarettes smokers, who wanted to quit smoking, were enrolled, out of which 104 (65.41%) were meeting the inclusion criteria and were randomly divided into the three experimental groups. 5 (14.71%) participants in the TMS & N group, 3 (9.09%) participants in the TMS & S group and 7 (18.92%) participants in the Sham group dropped out during the 5-day treatment period. The data of the participants who dropped out during the treatment (n=15) were removed from the final analysis.

# Supplementary Tables

| **sTable 1:** Means and standard deviations | | | | | | | | |  | | | |  | | | | |
| --- | --- | --- | --- | --- | --- | --- | --- | --- | --- | --- | --- | --- | --- | --- | --- | --- | --- |
| **Self-reported cigarette consumption** | | | | | | | | |  | | | |  | | | | |
|  | *Baseline* | | *After*  *Day 1* | | *After*  *Day 2* | *After*  *Day 3* | | *After*  *Day 4* | | | |  | | | |  |  |
| TMS&N group | 27.55(15.37) | | 8.72(8.97) | | 7.94(8.13) | 5.92(6.68) | | 5.27(7.51) | | | |  | | | |  |  |
| TMS&S group | 26.83(12.86) | | 8.67(8.56) | | 7.07(6.43) | 7.79(8.61) | | 4.67(6.50) | | | |  | | | |  |  |
| Sham group | 30.00(13.38) | | 10.60(7.49) | | 9.38(7.90) | 8.20(7.80) | | 6.27(7.84) | | | |  | | | |  |  |
| **CO (mean)** | | | | | | | | |  | | | |  | | | | |
|  | *Baseline* | | *Day 1* | | *Day 2* | *Day 3* | | *Day 4* | | | | *Day 5* | | | |  |  |
| TMS&N group | 23.64(14.68) | | 19.09(12.14) | | 11.09(9.69) | 9.98(10.15) | | 8.35(8.44) | | | | 8.98(9.68) | | | |  |  |
| TMS&S group | 17.24(10.17) | | 14.83(8.47) | | 9.94(8.17) | 9.61(6.63) | | 8.58(6.65) | | | | 8.03(7.27) | | | |  |  |
| Sham group | 21.13(9.63) | | 17.77(7.88) | | 11.03(7.50) | 8.85(6.58) | | 8.89(6.96) | | | | 8.18(7.02) | | | |  |  |
| **FTND** | | | | | | | | |  | | | |  | | | | |
|  | *Baseline* | | *End of treatment* | | *1-week follow up* |  | |  | | | |  | | | |  |  |
| TMS&N group | 5.86(2.26) | | 2.31(2.62) | | 1.79(1.55) |  | |  | | | |  | | | |  |  |
| TMS&S group | 6.03(2.37) | | 2.03(2.08) | | 2.04(2.49) |  | |  | | | |  | | | |  |  |
| Sham group | 6.77(2.27) | | 2.57(2.69) | | 2.64(2.41) |  | |  | | | |  | | | |  |  |
| **VAS (mean)** | | | | | | | | |  | | | |  | | | | |
|  | *Baseline* | | *Day 1* | | *Day 2* | *Day 3* | | *Day 4* | | | | *Day 5* | | | |  |  |
| TMS&N group | 24.21(30.64) | | 24.55(20.66) | | 15.62(16.69) | 12.45(15.33) | | 9.16(13.12) | | | | 10.57(15.74) | | | |  |  |
| TMS&S group | 30.87(28.63) | | 33.13(22.12) | | 23.54(18.88) | 22.32(19.87) | | 20.14(18.41) | | | | 16.08(16.14) | | | |  |  |
| Sham group | 25.70(30.42) | | 29.03(28.59) | | 21.87(24.12) | 17.48(21.78) | | 14.44(19.18) | | | | 10.46(13.13) | | | |  |  |
| **TCQ-SF** | | | | | | | | |  | | | |  | | | | |
|  | *Baseline* | | *End of treatment* | | *1-week follow up* |  | |  | | | |  | | | |  |  |
| TMS&N group | 45.38(17.95) | | 30.93(17.05) | | 29.84(14.18) |  | |  | | | |  | | | |  |  |
| TMS&S group | 52.03(16.20) | | 31.97(14.61) | | 32.17(21.57) |  | |  | | | |  | | | |  |  |
| Sham group | 43.83(17.25) | | 28.63(13.10) | | 27.28(14.82) |  | |  | | | |  | | | |  |  |
| **PSS-4** | | | | | | | | |  | | | |  | | | | |
|  | *Baseline* | | *End of treatment* | | *1-week follow up* |  | |  | | | |  | | | |  |  |
| TMS&N group | 5.97(2.18) | | 5.21(2.47) | | 4.42(2.59) |  | |  | | | |  | | | |  |  |
| TMS&S group | 6.53(2.40) | | 5.90(2.34) | | 6.00(2.66) |  | |  | | | |  | | | |  |  |
| Sham group | 5.60(1.98) | | 4.60(1.96) | | 4.76(2.35) |  | |  | | | |  | | | |  |  |
| **Motivation to quit smoking** | | | | | | | | | | |  | | | |  | | |
|  | *Baseline* | *End of treatment* | | *1-week follow up* | | |  | | |  | | | |  | | |  |
| TMS&N group | 77.78(21.18) | 82.41(20.59) | | 72.37(23.41) | | |  | | |  | | | |  | | |  |
| TMS&S group | 82.41(18.10) | 84.26(19.79) | | 77.17(30.07) | | |  | | |  | | | |  | | |  |
| Sham group | 80.83(20.43) | 80.83(23.38) | | 75.00(27.95) | | |  | | |  | | | |  | | |  |
| Data are means (standard deviation), averaged over all participants per group. | | | | | | | | | | | | | | | | | |

| **sTable 2:** Correlations between CO-measured and self-reported nicotine consumption | | | | |
| --- | --- | --- | --- | --- |
|  |  | **N** | ***r* value** | ***p* value** |
| CO Day 1 (mean) | Cigarettes smoked baseline | 89 | .469 | <.0001 |
| CO Day 2 (mean) | Cigarettes smoked after Day 1 | 89 | .756 | <.0001 |
| CO Day 3 (mean) | Cigarettes smoked after Day 2 | 86 | .643 | <.0001 |
| CO Day 4(mean) | Cigarettes smoked after Day 3 | 82 | .752 | <.0001 |
| CO Day 5 (mean) | Cigarettes smoked after Day 4 | 83 | .671 | <.0001 |
|  |  |  |  |  |
|  |  |  |  |  |

**References:**

1. MagVenture. Double Blinded Research Studies with MagPro. User Guide; 2018

2. Borckardt JJ, Nahas Z, Koola J, George MS. Estimating resting motor thresholds in transcranial magnetic stimulation research and practice: a computer simulation evaluation of best methods. *J ECT* (2006) 22:169-75. doi:10.1097/01.yct.0000235923.52741.72

3. Beam W, Borckardt JJ, Reeves ST, George MS. An efficient and accurate new method for locating the F3 position for prefrontal TMS applications. *Brain Stimul* (2009) 2:50-4. doi:10.1016/j.brs.2008.09.006
